# Vaccination with Omicron Inactivated Vaccine in Pre-vaccinated Mice Protects against SARS-CoV-2 Prototype and Omicron Variants

**DOI:** 10.3390/vaccines10071149

**Published:** 2022-07-19

**Authors:** Yuntao Zhang, Wenjie Tan, Zhiyong Lou, Yuxiu Zhao, Jin Zhang, Hongyang Liang, Na Li, Xiujuan Zhu, Ling Ding, Baoying Huang, Weimin Zhou, Yancen Guo, Zhaona Yang, Yuling Qiao, Zhenyu He, Bo Ma, Yao He, Di Zhu, Zhanhui Wang, Zhen Chang, Xue Zhao, Wei Wang, Ying Xu, Huiqin Zhu, Xiaotong Zheng, Chenlong Wang, Guangxue Xu, Guizhen Wu, Hui Wang, Xiaoming Yang

**Affiliations:** 1 Beijing Institute of Biological Products Company Limited, Beijing 100176, China; zhangyuntao@sinopharm.com (Y.Z.); zhao10306@126.com (Y.Z.); zhangjin_2566@sina.com (J.Z.); ayangyang@163.com (H.L.); lina1@sinopharm.com (N.L.); zhuxiujuan1@sinopharm.com (X.Z.); dinglingmail@163.com (L.D.); nancyguo1112@sina.com (Y.G.); yangzhaona2020@163.com (Z.Y.); lilian0601@163.com (Y.Q.); zhenyu_he09@126.com (Z.H.); mabo0303@163.com (B.M.); heyao142021@163.com (Y.H.); judy_bio@163.com (D.Z.); wangzhanhui2016@163.com (Z.W.); czhen9008@163.com (Z.C.); xiaoxueer012.student@sina.com (X.Z.); wangwei143@sinopharm.com (W.W.); xuying_beans@163.com (Y.X.); zhuhuiqin1@sinopharm.com (H.Z.); zhengxiaotong20@sina.com (X.Z.); 2 China National Biotec Group Company Limited, Beijing 100024, China; 3 National Institute for Viral Disease Control and Prevention, Chinese Center for Disease Control and Prevention (China CDC), Beijing 102206, China; tanwj@ivdc.chinacdc.cn (W.T.); baoying1233@163.com (B.H.); doglet44@163.com (W.Z.); 4 MOE Key Laboratory of Protein Science & Collaborative Innovation Center of Biotherapy, School of Medicine, Tsinghua University, Beijing 100084, China; louzy@mail.tsinghua.edu.cn (Z.L.); wangchenlong@mail.tsinghua.edu.cn (C.W.); xgx16@tsinghua.org.cn (G.X.)

**Keywords:** SARS-CoV-2, Omicron, inactivated vaccines, antibody

## Abstract

In response to the fast-waning immune response and the great threat of the Omicron variant of concern (VOC) to the public, we report the pilot-scale production of an inactivated Omicron vaccine candidate that induces high levels of neutralizing antibody titers to protect against the Omicron virus. Here, we demonstrate that the inactivated Omicron vaccine is safe and effective in recalling immune responses to the HB02, Omicron, and Delta viruses after one or two doses of BBIBP-CorV. In addition, the efficient productivity and good genetic stability of the manufactured inactivated vaccine is proved. These results support the further evaluation of the Omicron vaccine in a clinical trial.

## 1. Introduction

Variants of severe acute respiratory syndrome coronavirus 2 (SARS-CoV-2) have been identified following the first infection of coronavirus disease 2019 (COVID-19) in December 2019 [1]. The variant responsible for increased transmissibility, severe disease course, reduced effectiveness of treatments, and many other alarming factors is designated as the variant of concern (VOC), according to the Centers for Disease Control and Prevention (CDC) (CDC, 2021). A new, heavily mutated SARS-CoV-2 variant, B.1.1.529 (Omicron), was first reported to the World Health Organization (WHO) by South Africa on 23 November 2021, and then designated as a novel VOC on 26 November by WHO based on the subsequent and rapid increase in cases [2,3]. The Omicron genome contains 59 mutations, with 36 of these located in the spike protein, which serves as the host cell’s entrance medium and is the main target of neutralizing antibodies, and 15 mutations of Omicron located in the key receptor-binding domain (RBD) region. Previous research on SARS-CoV-2 variants revealed that the mutation on the RBD allowed the escape of vaccine-induced neutralizing antibodies, possibly due to the concentrated response of neutralizing antibodies to a limited set of RBD epitopes [4,5,6,7,8]. The above evidence suggested that the protection of vaccines against Omicron variants is reduced, which is a reminder that new vaccines for the Omicron variant need to be developed quickly.

The inactivated vaccine is widely utilized in preventing disease [9], and we had previously developed the BBIBP-CorV vaccine against SARS-CoV-2 (also described as HB02, wild-type COVID-19 virus) [10]. However, due to the frequent mutation of Omicron, the protective effect of the BBIBP-CorV vaccine is reduced. This study researched a candidate-inactivated Omicron vaccine to consider its safety and efficacy for preclinical performance.

## 2. Results

### 2.1. Vaccine Design and Production

**Figure 1:**
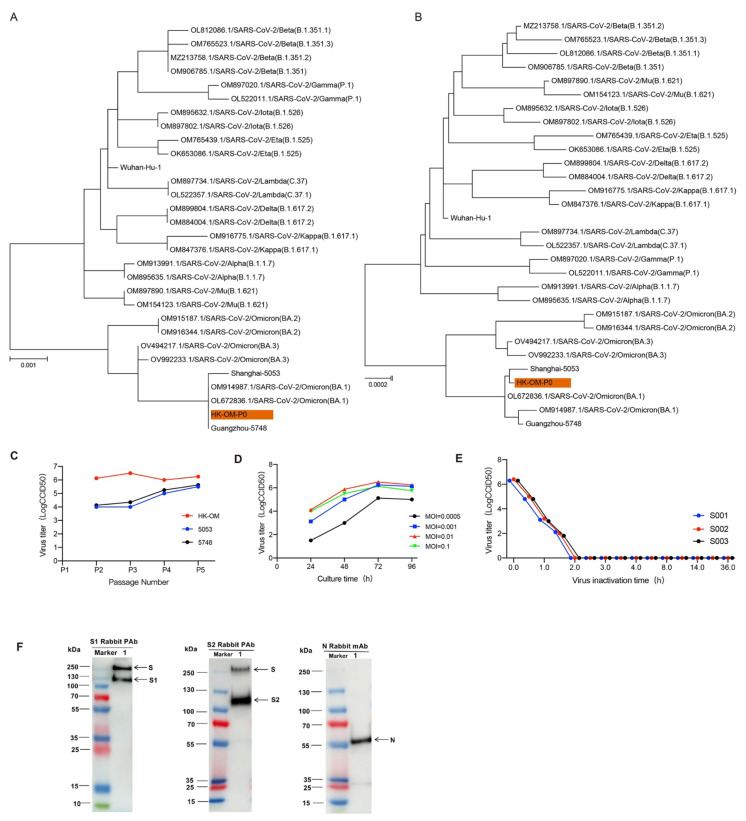
Characterization of the Omicron vaccine candidate. (**A**,**B**) SARS-CoV-2 maximum likelihood phylogenetic tree. (**C**) Growth kinetic analysis of 3 Omicron strains. (**D**) The effect of inoculation MOI on HK-OM-P7 stock virus titer. (**E**) Inactivation kinetics of three batches of virus supernatant. (**F**) The protein composition of Omicron vaccine (Vero cell) was evaluated by incubating with antibodies targeting S1 protein, S2 protein, and N protein.

To obtain the most suitable strain for vaccine production, we isolated three Omicron strains (BA.1 substrain) from throat swabs of patients from different regions and these three strains were named HK-OM-P0, 5748, and 5053. The three strains were scattered on a phylogenetic tree constructed from different pango lineages, suggesting that three strains were closely related to the BA.1 lineage of the Omicron strain (Figure 1A,B). Efficient proliferation and genetic stability are key features for the development of inactivated vaccines. By comparing the growth curves of three Omicron viruses, we found that HK-OM-P0 showed the fastest replication speed and the highest virus yields (Figure 1C). We chose the HK-OM-P0 strain for the further development of the inactivated Omicron vaccine. To investigate genetic stability, we serially cultured the virus in Vero cells to generate the P12 stock. The sequence of P12 stock showed 99.97% homology to the classic Omicron strain and there was no amino acid mutation in the N-terminal domain (NTD), receptor-binding domain (RBD), or furin cleavage site of the spike protein, indicating potential protection against the Omicron virus. As a result, the HK-OM-P0 strain was eventually used for the production of a subsequent vaccine.

Growth kinetic analysis of the P7 stock in Vero cells demonstrated that the stock virus could replicate efficiently and reach a peak titer of over 6.0 LgCCID_50_/mL within 72 h post-infection (hpi) at multiplicities of infection (MOI) of 0.001~0.01 (Figure 1D). The strain after adaptation for five generations was used as the original seed for vaccine production. To inactivate virus production, β-propionolactone was thoroughly mixed with the harvested viral solution at a ratio of 1:4000 at 2–8 °C. The inactivation of three batches of virus eliminated viral infectivity, validating the good stability and repeatability of the inactivation process (Figure 1E). Western blot analysis indicated that the vaccine stock contained viral protective antigens (S1 protein and S2 protein) and structural proteins (N protein) (Figure 1F).

### 2.2. Immunogenicity of the Omicron Inactivated Vaccine

**Figure 2:**
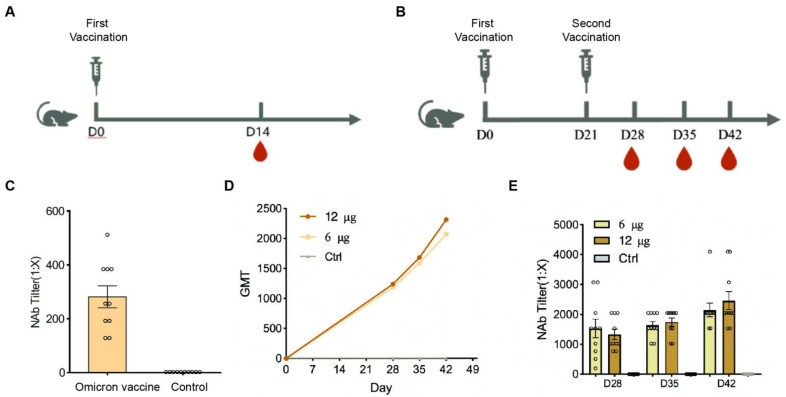
The immunogenicity of inactivated Omicron vaccine. (**A**) Mice model of Omicron immunogenicity. Mice were intramuscularly injected with a middle dose of the vaccine at day 0 (D0) and the NAb Tilter was tested at D14 of the vaccine against Omicron (*n* = 10/group). (**B**) Mice model of Omicron immunogenicity with middle (6 μg/dose) and high (12 μg/dose) dose twice on D0/D21, respectively. (**C**) The NAb Tilter of the vaccine against Omicron at D14 by the microtitration method. (**D**) The GMT level of the neutralizing antibody in each group. (**E**) The NAb Tilter against Omicron with middle (6 μg/dose) and high (12 μg/dose) doses of inactivated Omicron vaccine was performed at D28, D35, and D42 by the microtitration method (*n* = 10/group). Each circle represents the data of a mouse.

To assess the immunogenicity of Omicron, BALB/c mice were injected with different immunization programs and various doses (6 or 12 μg/dose) of vaccine mixed with aluminum hydroxide adjuvant (Figure 2A,B). Mice were intramuscularly injected with a middle (6 μg/dose) dose of the vaccine at day 0 (D0), and the neutralizing antibody titers (NAb Tilter) were tested after 14 days of the vaccine against Omicron. The results showed that the neutralization geometric mean titer (GMT) against Omicron was about 255, which indicates that the vaccine induced high levels of neutralizing antibodies in mice 14 days after immunization (Figure 2C). Furthermore, the mice were immunized twice on D0/D21 in the middle- and high-dose groups, respectively, and the serum was collected on D28, D35, and D42. NAb Tilter against Omicron was then performed. The results showed that the GMT to the Omicron virus presented an upward trend, and the induced GMT level reached a peak 21 days after the second immunization (D42) in both middle- and high-dose groups (Figure 2D), and there was no significant difference between the two groups (Figure 2D,E). This indicated that the Omicron inactivated vaccine had high immunogenicity against the Omicron virus.

### 2.3. Cellular Immune Response

**Figure 3:**
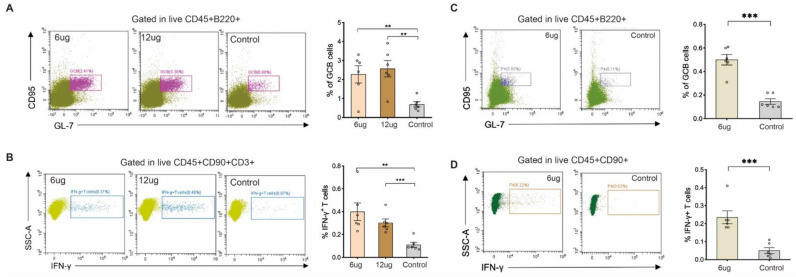
Cellular immune response of Omicron vaccine. (**A**) The percentage of GCB cells was analyzed in mice immunized with Omicron vaccine after 28 days. (**B**) The percentage of IFN-γ expressing T cell was analyzed in mice immunized with Omicron vaccine after 28 days. (**C**) The percentage of GCB cells was analyzed in mice immunized with Omicron vaccine after 42 days. (**D**) The percentage of IFN-γ expressing T cells was analyzed in mice immunized with Omicron vaccine after 42 days. Each circle represents the data of a mouse. Error bars represent SEM. ** *p* < 0.01; *** *p* < 0.001.

To explore the effect of the Omicron vaccine on cellular immunity, we isolated the immune cells from the mouse spleen, which were immunized with the middle- and high-doses of the Omicron vaccine. Then, we analyzed the germinal center B cell (GCB) and T cell immune responses. The results showed that, compared with the control group, the percentage of GCB cells in the middle- and high-dose groups was significantly increased (Figure 3A,B), and the secretion of IFN-γ in T cells was also induced after immunization (Figure 3C,D). This suggests that the Omicron inactivated vaccine could induce cellular immune responses in addition to humoral immune responses. To confirm this fact, the mice were also immunized with the middle dose of the Omicron vaccine twice and we analyzed the spleen immune cells on D42 by flow cytometry. Consistent with 7 days after the second immunization, the percentage of GCB cells and IFN-γ expressing T cells was elevated after two doses of immunization (Figure 3E,F). After two doses of immunization, the Omicron vaccine could effectively promote the humoral immune response and cellular immune response, which indicated that the candidate vaccine had a strong protective effect on the Omicron virus.

### 2.4. Heterologous Vaccination of Boosting Strategies

**Figure 4:**
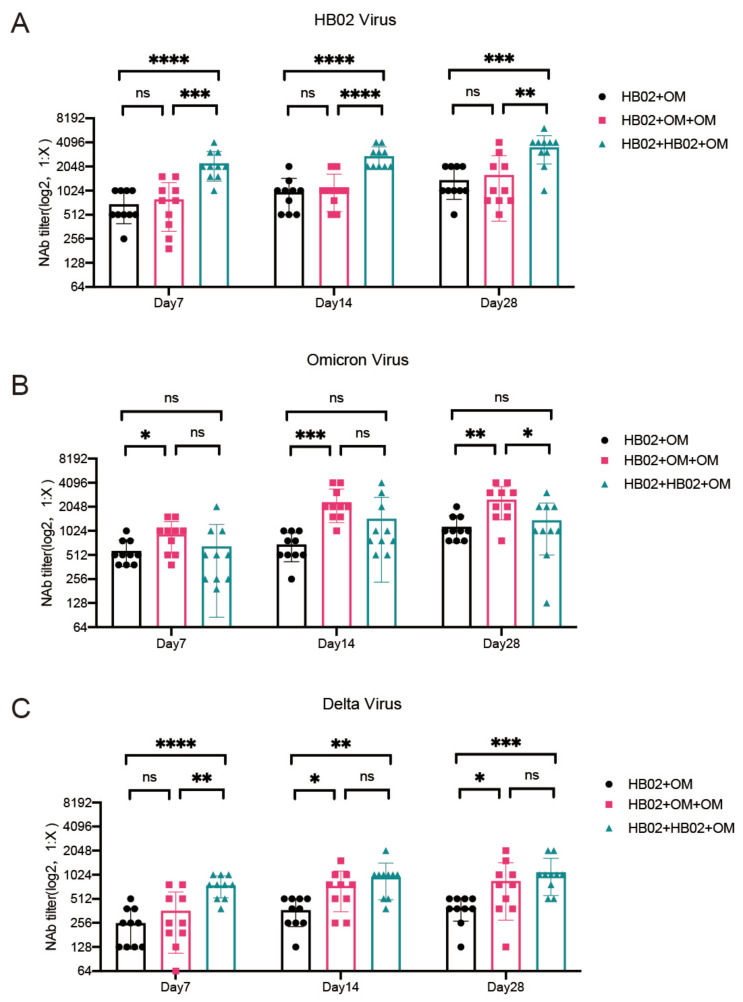
The neutralizing antibody tilters for Delta viruses after three types of sequential immunization. (**A**) The neutralizing antibody tilters against HB02 virus in serum of mice at days 7/14/28 after the last dose of vaccine, with sequential immunization. (**B**) The neutralizing antibody tilters against Omicron virus in serum of mice at days 7/14/28 after the last dose of vaccine, with sequential immunization. (**C**) The neutralizing antibody tilters against Delta virus in serum of mice at days 7/14/28 after the last dose of vaccine, with sequential immunization. Error bar represents SEM from eight independent experiments (*t*-test, * *p* < 0.05, ** *p* < 0.01, *** *p* < 0.001, **** *p* < 0.0001).

Globally, SARS-CoV-2 continues to circulate in waves, necessitating several immunizations. As a result, it is critical to understand the impact of earlier vaccinations on subsequent boosters. Based on the fact that 90% of the population in China has received at least a dose of inactivated vaccine, we designed three types of sequential immunizations (HB02 + Omicron; HB02 + Omicron + Omicron; HB02 + HB02 + Omicron) with a HB02 and Omicron inactivated vaccine to study the neutralizing antibody response. Blood was regularly collected from the fundus vein 7, 14, and 28 days after the last dose of immunization, and then a serum neutralizing antibody was detected. We found all three types of sequential immunizations could stimulate the NAb response to the HB02 and Omicron strains (Figure 4A,B). However, it seems that the HB02 and Omicron inactivated vaccine could not increase the neutralizing antibodies’ titer to each other as there was no significant difference in NAb Titer compared to the other virus strain with one more dose of inactivated vaccine (Figure 4A,B). Sequential immunization could also stimulate the NAb response to the Delta strain, and the trend of NAb was increased for an indicated time after sequential immunization (Figure 4C). Furthermore, the NAb response to Delta was significantly lower than to HB02 or Omicron 28 days after immunization as expected (Figure S1). Moreover, a third dose of the vaccine could further improve the neutralization level of the Delta strain significantly (Figure 4C). These data suggested that sequential immunization with Omicron inactivated vaccine may play a broad-spectrum protective role against COVID-19 VOCs.

### 2.5. Safety

**Figure 5:**
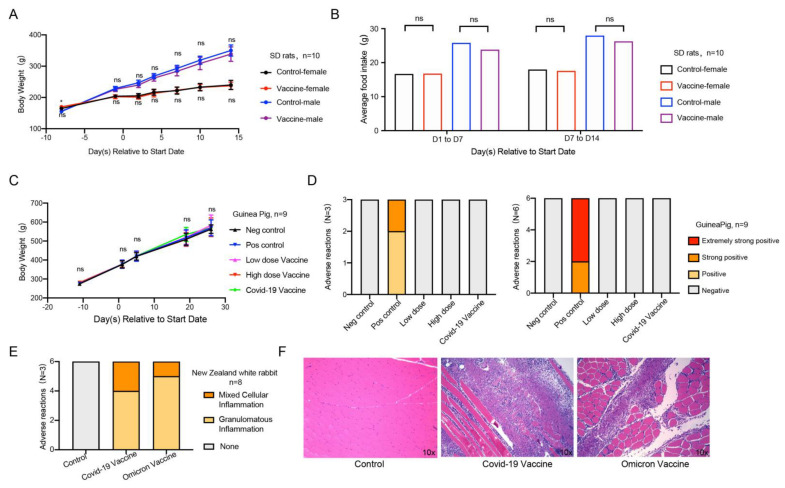
Safety evaluation of Omicron vaccine. (**A**,**B**) Acute toxicity experiment. Effects of acute toxicity on body weight and food intake of SD rats, *n* = 10 in each group, 5/gender/group. (**C**,**D**) Active allergy experiment. Effect of systemic active allergy experiment on the body weight of guinea pigs. Incidence of adverse reactions in active systemic hypersensitivity test 14 days and 21 days after the last sensitization of Omicron vaccine, and *n* = 9 in each group. (**E**,**F**) Muscle stimulation experiment. Statistical chart of adverse reactions of New Zealand white rabbits in muscle stimulation experiment, and (**F**) histopathological sections of adverse reactions, *n* = 8 in each group. Error bars represent SEM. * *p* < 0.05.

To evaluate the safety of the Omicron vaccine, we performed acute toxicity, systemic anaphylaxis, and muscle stimulation experiments. In the acute toxicity experiment, we randomly divided female and male SD rats into two groups with similar body weights. The negative control group was intramuscularly injected with sodium chloride solution, and the experimental group was intramuscularly injected with a middle (6 μg/dose) dose of inactivated vaccine. We observed and recorded adverse reactions regularly. The results appeared to show that no death or dying was found in each group of animals during the test, and no abnormal reaction was found in the clinical observation. Compared with the negative control group of the same gender, the weight (Figure 5A) and food intake (Figure 5B) of the animals in the experimental group were similar. The results of the anatomical observation of rats showed that there were no abnormal changes in the main organs and tissues in each group. The above results indicated that the Omicron vaccine did not induce acute toxicity in rats.

To evaluate the effect of the Omicron vaccine on immediate anaphylaxis in guinea pigs, we injected the negative control, positive control, low-dose vaccine, high-dose vaccine, and commercially available control vaccines by intramuscular injection three times to simulate sensitization at D1/D3/D5. At 14 days (D19) and 21 days (D26) after sensitization, the animals in each group were challenged with an intravenous injection to observe whether the animals in each group developed allergic reaction symptoms. The results showed that the weight change of guinea pigs in the five groups was similar (Figure 5C). In addition to the allergic reaction of guinea pigs in the positive control group, the other four groups of guinea pigs did not have an allergic reaction on D19 (Figure 5D left) or D26 (Figure 5D right), suggesting that the inactivated vaccine of Omicron is safe on sensitization.

To evaluate the irritation of the Omicron vaccine, we used the New Zealand white rabbit model by injecting the vaccines into the lateral muscles of the hind limbs three times (once every 7 days). We evaluated the stimulation level regularly, recorded the adverse reactions, and carried out a histopathological examination. The results showed that, three days after the last injection (D18), similar pathological changes were observed in the local administration of the positive control vaccine group and Omicron vaccine group. The adverse reactions presented granulomatous inflammation and mixed cellular inflammation, which is related to the administration method of injection, and the incidence and level of adverse reactions were consistent with the COVID-19 vaccine (Figure 5E). Fourteen days (D29) after the last administration, slight granulomatous inflammation and slight bleeding were still seen in the administration part of the commercial control and test article, indicating that the irritant response did not fully recover (Figure 5F). The adverse response of the administration tissue was recovered after prolonged observation.

## 3. Discussion

The SARS-CoV-2 variant of the Omicron strain spread rapidly once it appeared. The development of vaccines against the Omicron variant with high immunogenicity and safety is crucial for the control of the global COVID-19 pandemic and the prevention of further illness and fatalities. Here, we report the pilot-scale production of an inactivated Omicron vaccine candidate that induces high levels of neutralizing antibody titers to provide protection against the Omicron virus and provide a heterologous vaccination with boosting strategies based on one/two doses of HB02 vaccine, which might have a better protective efficacy against COVID-19 VOCs.

Before our study, many vaccines against SARS-CoV-2 were reported; however, a large number of mutations in the Omicron variant, especially in the receptor-binding domain (RBD) and the N-terminal domain (NTD) of the spike protein, allowed the escape of vaccine-induced neutralizing antibodies, which resulted in a significant decrease in the protective efficacy of vaccines against the Omicron variant. The protective effect of serums from 15 weeks after two doses of ChAdOx1-S (Vaxzevria, AstraZeneca) against Omicron is close to 0 [11]. Vaccine effectiveness decreases by 11.4-fold against Omicron compared with a wild type at 6 months following the second dose of Pfizer BNT162b2 (Pfizer-BioNTech, Comirnaty^®^) [12]. Furthermore, there is a 20-fold reduction in neutralizing ability compared to D614G 6 months after two doses of mRNA-1273 (Spikevax, Moderna) [13]. Based on these studies, it is necessary to develop a vaccine against the Omicron variant. In our study, a two-dose immunization with middle (6 μg) and high (12 μg) doses of inactivated Omicron vaccine conferred high immunogenicity against the Omicron virus and promoted the production of high levels of neutralizing antibodies in mice. The safety of the vaccine has been demonstrated in animal models of rats, guinea pigs, and New Zealand white rabbits.

It is worth mentioning that a large number of people have accepted SARS-CoV-2 vaccinations, implying that a heterologous vaccination with boosting strategies is a good technique to defend against the prototype and variant strains. However, there is currently little evidence to support heterologous vaccination [14]. Heterologous administration was reported to have strong immunogenicity and acceptable reactogenicity in a systematic review, which indicates that a heterologous vaccination is a plausible and feasible strategy to prevent COVID-19 [15]. A recent study showed that using all four vaccines as a third dose is safe and results in a stronger immune response [16]. Our study shows that, after one or two doses of BBIBP-CorV, the Omicron vaccine is safe and effective in recalling immune responses to both HB02 and the Omicron virus regardless of NAb titer or cellular immunity, and the heterologous vaccination strategy appears to be better. These strategies, meanwhile, could stimulate the NAb response to the Delta virus and might perform a protective role. This is a commendable effort, and provides new evidence for the feasibility of a heterologous vaccination strategy, but further research is needed to validate the benefits and determine the best combinations, doses, and intervals.

In addition to neutralizing antibodies, 2019-nCoV can also induce a cellular immune response, and the levels of 2019-nCoV-specific CD4+ and CD8+ T cells are associated with mild symptoms after infection [17], suggesting the protective role of T cell immunity in 2019-nCoV infection. It has been reported that the majority of the recovered COVID-19 patients still have T cell memory one year after infection, which represents how cellular immunity plays an important role in COVID-19 infection [18]. Studies of patients infected with SARS-CoV in 2003 found that the virus caused long-lasting T cell responses that lasted for 6 years [19]. T cells were demonstrated to recognize peptides derived from the viral spike, nucleoprotein, and matrix, as well as other viral proteins, and specific CD4+ T cells are required for evoking powerful B cell responses that lead to antibody affinity maturation [20]. Unlike other kinds of vaccines, inactivated vaccines do not deliver a single antigen. It is feasible to produce more effective cellular immunity in addition to antibody protection. Considering that T cells respond to the entire spike protein, a few mutations are less likely to affect them, meaning that inactivated vaccinations might provide better protective efficacy against mutated viruses. However, it is unclear whether the inactivated virus can be protected through other mechanisms, and more research is needed.

In the face of the serious challenge to current antibodies and vaccines posed by SARS-CoV-2 variant mutations, the development of the Omicron inactivated vaccine and a heterologous vaccination strategy provides a potential solution to the COVID-19 pandemic. Clinical trials are expected to commence based on the findings presented here.

## 4. Animal Models

Rats, mice, Hartley guinea pigs, and New Zealand rabbits were purchased from Beijing weitonglihua Experimental Animal Technology Co., Ltd. All animals participating in this research were in good health and were not involved in other experimental procedures. All animals were allowed free access to water and diet and provided with a 12 h light/dark cycle (temperature: 18–28 °C, humidity: 40–70%). The mice, guinea pigs, rabbits, and rats were bred and maintained in a specific pathogen-free (SPF) environment at the Laboratory Animal Center of Beijing institute of biological products Co., Ltd.

## 5. Virus

The Omicron virus Shanghai-5053 (shortened as 5053), Guangzhou-5748 (shortened as 5748), and HK-OM-P0 were used as the vaccine candidate. For the neutralization assay, P7 stock of prototype virus 19nCoV-CDC-Tan-HB02 (shortened as HB02), HK-OM-P0, and nCoV210077 (Delta) was used.

## 6. Method Details

### 6.1. Phylogenic Tree Analysis

Genome sequences and spike gene sequences for SAR-CoV-2 were retrieved from NCBI (https://www.ncbi.nlm.nih.gov/nucleotide/, accessed on 31 December 2021), and 30 sequences were obtained from different pango lineages. Multiple sequence alignment and phylogenetic reconstruction were performed by using MEGA6 (http://mega6.software.informer.com/, accessed on 10 June 2022).

### 6.2. Growth Kinetics Curve

To measure the growth kinetics, three Omicron viruses (HK-OM-P0, 5748, and 5053) separated from the patients were harvested 48–72 h in Vero cells after inoculation with an MOI of 0.001–0.01. Then, the titer was calculated by the Karber method based on a microdose cytopathogenic efficiency (CPE) assay, as described before [21]. The virus was serially cultured in Vero cells to generate the P5 stock and the titer of each stock was calculated. HK-OM-P0 was incubated with Vero cell monolayers with an MOI of 0.0005, 0.001, 0.01, and 0.1. The cells were then cultured in a 5% CO_2_ incubator at 37 °C for 96 h, and the supernatant was taken every 24 h for virus titer determination. The curve was plotted with Excel software.

### 6.3. Validation of the Inactivation

The effective inactivation of the virus was validated in a sample from three batches of Omicron COVID-19 vaccines (Vero cell). Ten milliliters of inactivated Omicron was used to inoculate Vero monolayers in 75 cm^2^ flasks, and the cells were then cultured in a 5% CO_2_ incubator at 37 °C for 4 days. The supernatant was taken every half hour for virus titer determination. The virus titer was determined by a microdose cytopathogenic efficiency (CPE) assay. Serial 10-fold dilutions of virus-containing samples were mixed with 3~5 × 10^4^ Vero cells and then plated in 96-well culture plates. After 4 days of culture in a 5% CO_2_ incubator at 37 °C, cells were checked for the presence of a CPE under a microscope. The virus titer was calculated by the Karber method [21]. The curve was plotted with Excel software.

### 6.4. Western Blotting

Samples containing 45 μg of protein were mixed with loading buffer and then boiled at 95 °C for 10 min. The proteins were separated by 8% sodium dodecyl sulfate polyacrylamide gel electrophoresis (SDS-PAGE) and transferred onto a polyvinylidene fluoride (PVDF) membrane (300 mA, 2.5 h). The membrane was sealed in phosphate-buffered saline with Tween-20 (PBST) with 5% skim milk at 25 °C for 2 h. This was subsequently incubated overnight with the primary antibodies anti-S1 protein rabbit polyclonal Ab (Sino Biological, Beijing, China) (1:1000 dilution) or anti-S2 protein rabbit polyclonal Ab (Sino Biological) (1:1000 dilution) or anti-N protein rabbit monoclonal Ab (Sino Biological, Beijing, China) (1:1000 dilution) at 25 °C. The membrane was incubated for 1 h at room temperature with the secondary antibodies goat anti-rabbit IgG H&L (HRP) (GE NA934, 1:2000). Protein bands were visualized using enhanced chemiluminescence (Cytiva, Marlborough, MA, USA).

### 6.5. Vaccine Preparation

HK-OM- P7 viruses were cultured in a 10 L basket bioreactor at a temperature of 36 ± 1 °C. The virus solution was harvested 48–72 h after inoculation and was then inactivated with b-propiolactone at a ratio of 1:4000 at 2–8 °C for 20–24 h, followed by chromatography purification. The final bulk was prepared by adding aluminum hydroxide as the adjuvant and dilution buffer containing phosphate.

### 6.6. Vaccine Immunogenicity Analysis and Neutralization Assay

Mice were randomly divided into different groups and intramuscularly immunized with the Omicron vaccine for one or two doses. Blood was collected from each group before immunization, and the serum was isolated as a control. Each mouse was injected with 0.5 mL of the sample. The neutralization assay was based on the microplate CPE (micro-cytopathogenic efficiency) method. Briefly, the serum to be tested was diluted by a 2-fold series, starting with a dilution ratio of 1:4. Then, the virus was added to each plate and incubated for 2 h in a 37 °C incubator to initiate neutralization. Cell suspension was added and incubated for another 4 days, followed by observing the CPE. The detailed protocol was described previously [21].

### 6.7. T Cell Response Assay

For lymphocytes analysis in the spleen, the spleen was minced, ground with an injection syringe, and then red blood cells were removed by RBC (Biolegend, USA, 420301). The cells were filtered before use. Immune cells were seeded on 96-well plates at a density of 1 × 10^6^ and stimulated with inactivated virus stock solution (8 μg/well) in DMEM medium (GIBCO, Waltham, MA, USA, C11995500BT) with 1% penicillin–streptomycin (Procell, Las Vegas, NV, USA, PB180120) and 10% FBS (GIBCO, USA, 10099141) for 10 h, and BFA (Biolegend, San Diego, CA, USA, 420601) for 4 h. The diluent buffer for flow detection antibody was pbsf (PBS + 1% FBS). Dead cells were excluded by the Fixable Viability Dye eFluor 506. GCB cells were presented by GL-7+ (Biolegend, USA, 144603) CD95+ (BD, Franklin Lakes, NJ, USA, 561985) and gated in live CD45+ (Biolegend, USA, 103126) B220+ (BioLegend, USA, 103232). IFN-γ+ T cells were presented by IFN-γ+ (eBioscience, 17-7311-82) and gated in live CD45+ CD90+ (BioLegend, USA, 105306). Flow cytometry was performed on CytoFLEX S instruments (Beckman, Brea, CA, USA) and analyzed with CytoFLEX S FlowJo software. The dosages of the reagents used are in Table 1.

### 6.8. Acute Toxicity

Twenty rats (10/gender) were divided into 4 groups (5/gender/group) and intramuscularly injected with 6 μg of Omicron vaccine or sodium chloride as a control. The body weight and food intake were recorded.

### 6.9. Allergy Study

Thirty-six male guinea pigs were evenly divided into 5 groups. Then, 1/5 were injected with sodium chloride as a negative control, and 1/5 were injected with 20 mg (0.5 mL) of human blood albumin as a positive control. The low-dose group was injected with 0.05 mL (1.5 μg) vaccine, the high-dose group was injected with 0.5 mL (12 μg) vaccine. The commercial vaccine group was injected with 0.5 mL (12 μg) HB02 vaccine. Sensitization was carried out on D1, D3, and D5 by intramuscular injection. Three guinea pigs from each group were selected for intravenous excitation via the foot at D19, and second excitations were performed on the remainder of the guinea pigs in each group, at D19 and D26.

### 6.10. Muscle Stimulation Study

The muscle stimulation experiment’s animal model was the New Zealand white rabbit, with *n* = 8 in each group, in which half of the males and half of the females were intramuscularly injected to provide the Omicron or control vaccines three times on D1, D8, and D15. Two were euthanized at 3 days (D18), and the remaining were euthanized 14 days (D29) after the last dose. The local tissues of animals were observed and analyzed by immunohistochemistry.

## Figures and Tables

**Table 1:** The dosage of the reagents.

Reagent	Dosage
Purified anti-mouse CD16/32 Antibody	0.125 μg/50 μL
BV421 anti-mouse CD45 Antibody	0.05 μg/50 μL
PE/Cyanine7 anti-mouse CD3ε Antibody	0.05 μg/50 μL
FITC anti-mouse CD90.2 Antibody	0.05 μg/50 μL
AF700 anti-mouse/human CD45R/B220 Antibody	0.05 μg/50 μL
Super Bright 600 anti-mouse CD4 Antibody	0.05 μg/50 μL
Fixable Viability Dye eFluor™ 506	1000×
FITC anti-mouse GL-7 Antibody	0.1 μg/50 μL
APC anti-mouse IFN-γ Antibody	0.15 μg/50 μL
PE anti-mouse CD95 Antibody	0.1 μg/50 μL

## Data Availability

Not applicable.

## Supplementary Materials

The following supporting information can be downloaded at: https://www.mdpi.com/article/10.3390/vaccines10071149/s1, Figure S1: The neutralizing antibody tilters for different viruses after three types of sequential immunization; Figure S2: The flow graph of the GCB and IFN-γ+ T cells.
