# Supplementary material for: Vaccination with Omicron Inactivated Vaccine in Pre-vaccinated Mice Protects against SARS-CoV-2 Prototype and Omicron Variants
Source: Vaccines (Basel). 2022 Jul 19;10(7):1149. doi: 10.3390/vaccines10071149 (PMC9318641; doi:10.3390/vaccines10071149)
Supplement: Supplementary file 1 [file vaccines-10-01149-s001.zip › vaccines-1795033-supplementary.pdf]

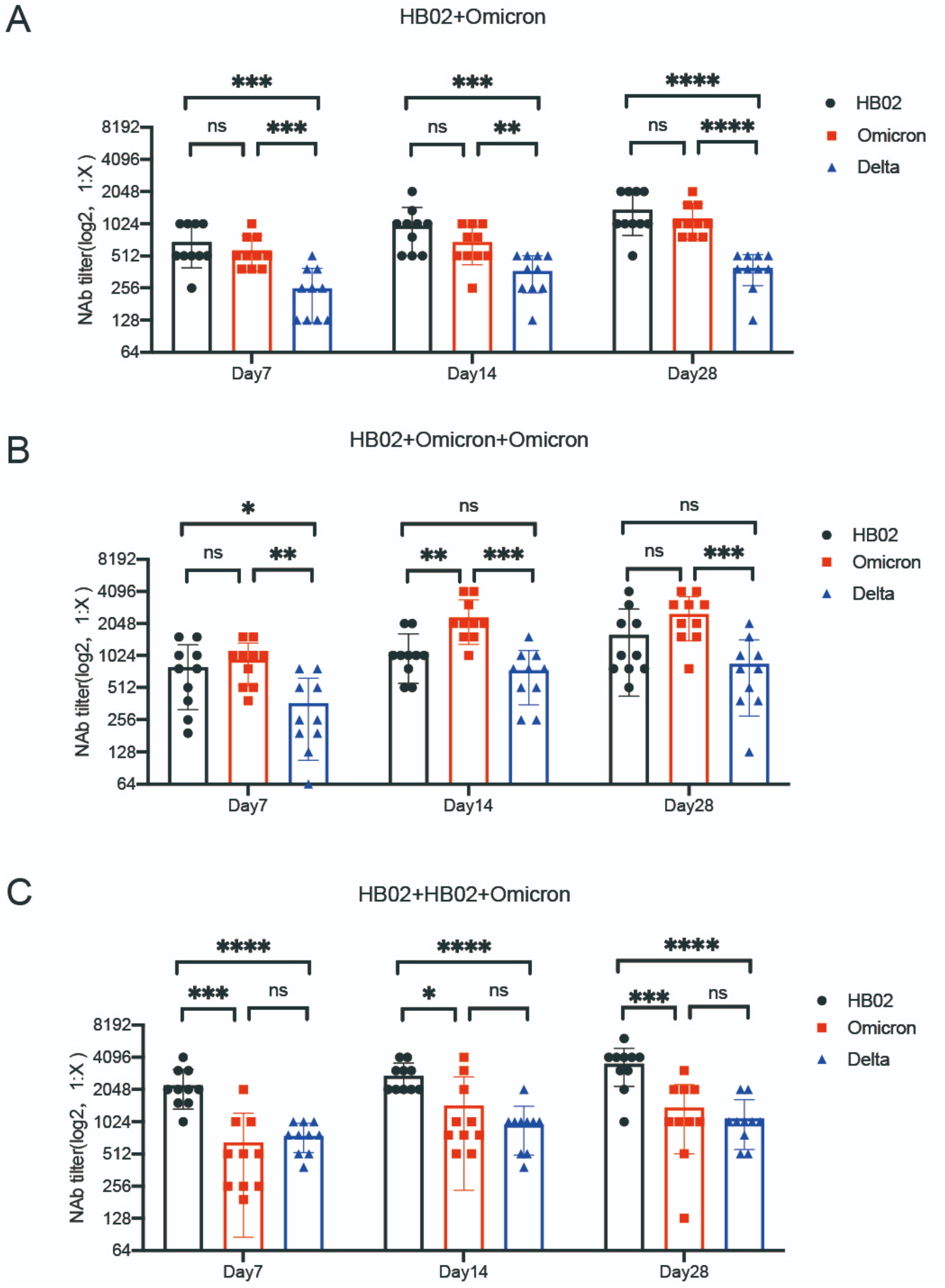

Figure S1. The neutralizing antibody tilters for different viruses after three types of sequential immunization.
(**A**) The neutralizing antibody tilters against HB02, delta, omicron strains in serum of mice at day7/14/28 with sequential immunization of HB02+Omicron inactivated vaccines.
(**B**) The neutralizing antibody tilters against HB02, delta, omicron strains in serum of mice at day7/14/28 with sequential immunization of HB02+Omicron+Omicron inactivated vaccines.
(**C**) The neutralizing antibody tilters against HB02, delta, omicron strains in serum of mice at day7/14/28 with sequential immunization of HB02+HB02+Omicron inactivated vaccines.
Error bar represents SD from eight independent experiments (*t* test, * $p < 0.05$, ** $p < 0.01$, *** $p < 0.001$, **** $p < 0.0001$).

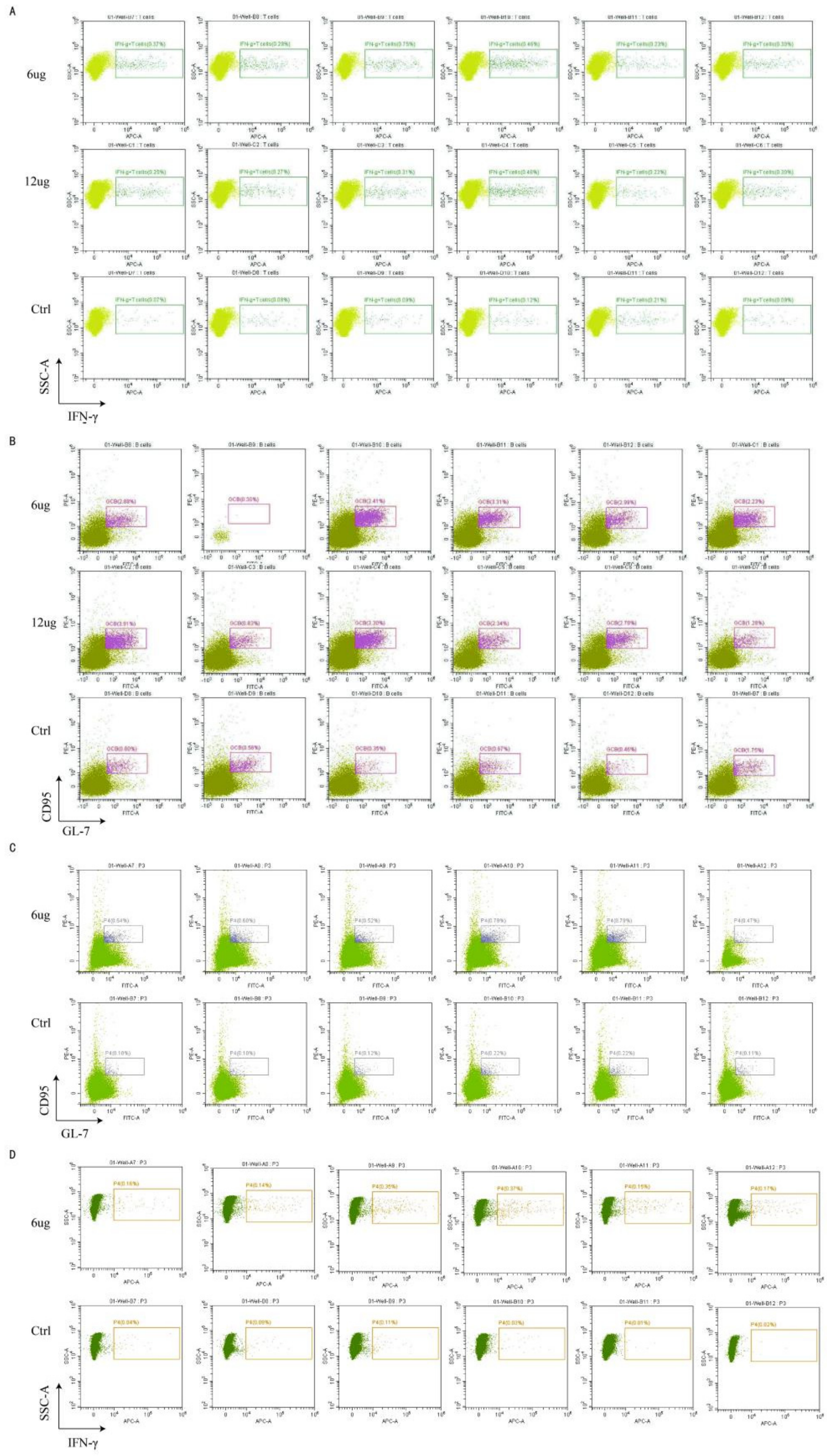

Figure S2. The flow graph of the GCB and IFN-γ+ T cells.

(**A**) The percentage of IFN-γ expressing T cells was analyzed in mice immunized with Omicron vaccine after 28 days. (**B**) The percentage of GCB cells was analyzed in mice immunized with Omicron vaccine after 28 days. (**C**) The percentage of GCB cells was analyzed in mice immunized with Omicron vaccine after 42 days. (**D**) The percentage of IFN-γ expressing T cells was analyzed in mice immunized with Omicron vaccine after 42 days.
